# Spontaneous Ischemic Cholecystitis in a Patient with Hereditary Hemorrhagic Telangiectasia (HHT)

**DOI:** 10.3390/jcm13226653

**Published:** 2024-11-06

**Authors:** Romain L’Huillier, Alexandre Garnaud, Olivier Monneuse

**Affiliations:** 1 Department of Medical Imaging, Edouard Herriot Hospital, Hospices Civils de Lyon, 69002 Lyon, France; alexandregarnaud16@gmail.com; 2 LabTAU, INSERM U1032, 69003 Lyon, France; 3 Everest, The French Comprehensive Liver Center, Hospices Civils de Lyon, University of Lyon, 69002 Lyon, France; 4 Department of Digestive Surgery and Emergency Surgery, Edouard Herriot Hospital, Hospices Civils de Lyon, 69002 Lyon, France; olivier.monneuse@chu-lyon.fr

**Keywords:** ischemic cholecystitis, hereditary hemorrhagic telangiectasia, arteriovenous malformation, biliary ischemia, ischemic cholangitis, telangectases

## Abstract

**Background/Objectives:** Hereditary hemorrhagic telangiectasia (HHT) is an autosomal dominant disorder characterized by abnormal blood vessel formation, leading to recurrent epistaxis, cutaneous and mucosal telangiectases, and visceral arteriovenous malformations (AVMs). Hepatic involvement may result in complications such as high-output heart failure, portal hypertension, and biliary ischemia. We report an uncommon case of ischemic cholecystitis in a patient with HHT. **Methods:** A 57-year-old male with HHT type 1, including gastric telangiectases and hepatic AVMs, presented with anemia, melena, epigastric pain, and a history of recurrent epistaxis. Imaging revealed gastric telangiectases and liver AVMs, consistent with HHT. Following an episode of severe epistaxis and aspiration pneumonia, the patient developed right upper quadrant pain. **Results:** Abdominal CT and ultrasound identified thickening of the gallbladder wall, segmental enhancement defects, and a perivesicular fluid effusion, suggestive of acalculous cholecystitis. A laparoscopic cholecystectomy was performed, revealing ischemic cholecystitis with necrotic gallbladder walls. **Conclusions:** This case underscores the potential for ischemic cholecystitis in patients with HHT and liver involvement, particularly under conditions of acute hemodynamic instability. Clinicians should be vigilant in recognizing this rare complication, especially in patients with established HHT and associated hepatic vascular anomalies.

## 1. Background

Hereditary hemorrhagic telangiectasia (HHT) also known as Osler–Weber–Rendu syndrome is an autosomal dominant disease that causes highly vulnerable abnormal blood vessel formation [1]. It is clinically characterized by recurrent spontaneous epistaxis, cutaneous, mucosal telangiectases, and visceral arteriovenous malformations (AVMs). The reported prevalence of HHT ranges between 1:5000 and 1:8000 by region [2].

Clinical diagnosis is based on the Curaçao criteria (recurrent epistaxis, telangiectases, visceral arteriovenous malformations, and family history) and is considered definite if three criteria are fulfilled [3].

In 90% of patients with a definite clinical diagnosis of HHT, a mutation is identified in one of these three genes: endoglin (ENG, HHT type 1), activin receptor-like kinase-1 (ACVRL1, HHT type 2), and Mothers against decapentaplegic homolog 4 (SMAD4, juvenile polyposis–HHT overlap syndrome) [4].

The lesions that result from the genetic mutations in HHT may range in size from very small telangiectases to several-centimeter-diameter AVMs.

AVMs most often involve the lungs, liver, gastrointestinal tract and brain [3].

Given the dual blood supply to the liver, three types of shunts may be found: arteriovenous (hepatic artery to hepatic vein, the most frequent), arterioportal (hepatic artery to portal vein), and portovenous (portal vein to hepatic vein) [5]. Symptoms occur in about 8% of the patients with liver involvement [6,7,8] which may result in portal hypertension (in the case of arterioportal shunt), hepatic encephalopathy (in the case of portovenous shunt), high-output heart failure (in the case of arteriovenous or portovenous shunt), and biliary ischemia [9,10] which can induce biliary necrosis and liver abscesses [11] due to a blood flow steal caused by arteriovenous shunts.

We report here a case of ischemic cholecystitis in a 57-year-old patient with HHT.

## 2. Case Presentation

A 57-year-old patient with HHT type 1 followed at the French National HHT Reference center (Hôpital Femme-Mère-Enfant, Hospices Civils de Lyon, France) was hospitalized for blood transfusion and examination via gastroduodenoscopy and colonoscopy. The patient suffered from anemia, diarrhea associated with melena, and epigastric pain for the past 6 weeks with a negative infectious workup. There was no nasal or cutaneous bleeding and the neurological examination was perfectly normal.

**Figure 1:**
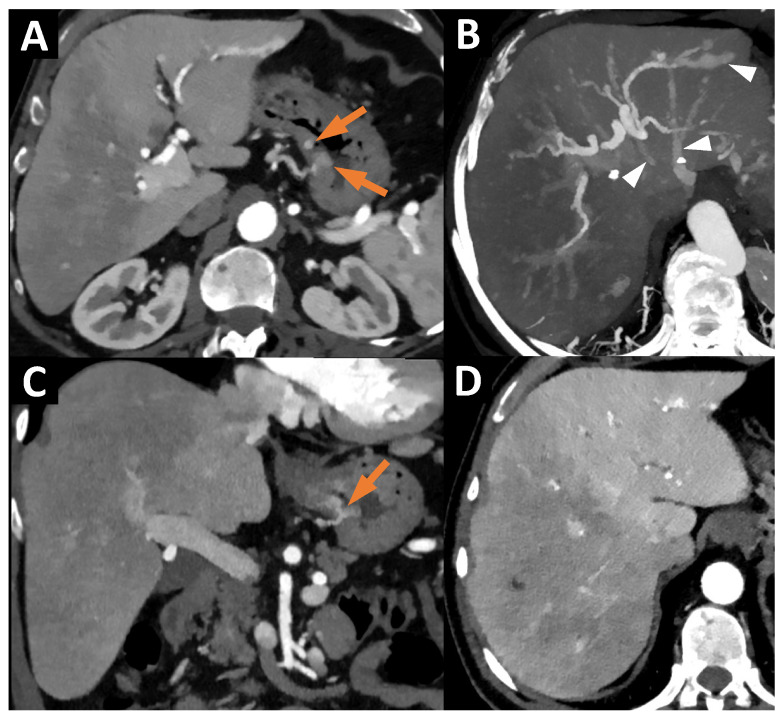
(**A**) Axial CT at the arterial phase (40 keV reconstruction), showing telangiectases of the stomach body (orange arrows). (**B**) Axial CT at the arterial phase, with Maximum Intensity Projection (MIP) 25 mm, showing a voluminous arteriovenous shunt with early enhancement of the left hepatic vein (white arrowheads). (**C**) Coronal CT at the arterial phase (40 keV reconstruction), showing telangiectases of the stomach body (orange arrow). (**D**) Axial CT at the arterial phase (40 keV reconstruction), showing heterogeneous hepatic enhancement linked to the arteriovenous shunt between the left branch of the hepatic artery and the left hepatic vein.

An abdominopelvic computed tomography (CT) scan performed in July 2024 did not show tumoral or infectious causes but found HT-related lesions (gastric telangiectases as well as liver AVMs) (Figure 1).

Among his past medical history, we found cutaneous telangectases, diffuse telangectases in the gastrointestinal tract and in the nasal fossae causing chronic anemia due to hemorrhages from them, a pulmonary AVM which was embolized in 2005 and 2022, liver AVMs, and post-smoking chronic obstructive pulmonary disease with emphysema. His treatment with bevacizumab, initiated in November 2022, was stopped in June 2024 due to the lack of clinical improvement.

The latest transthoracic ultrasound performed did not show any cardiac failure, with a normal cardiac index and normal pulmonary artery pressure.

After the endoscopy, which allowed the treatment of gastric telangectases, the patient was transferred to the intensive care unit with acute respiratory failure due to aspiration pneumonia after an episode of severe epistaxis. The patient was quickly extubated after minimal support with catecholamines, and an antibiotic treatment was started.

The day after, the patient suffered from abdominal pain in the right upper quadrant, requiring morphine. On clinical examination, there was tenderness on palpation in the region of the liver, increased on deep inspiration; no abdominal mass was palpated.

Biological tests revealed a biological inflammatory syndrome (C-reactive protein at 185 mg/L), and hepatic cytolysis (aspartate amino transferase (AST) at 200 U/L and alanine amino transferase (ALT) at 216 U/L without cholestasis (normal serum levels of Gamma Glutamyl Transferase (GGT) and conjugated bilirubin).

An abdomino-pelvic CT was performed to explore the pain in the right upper quadrant of the abdomen.

**Figure 2:**
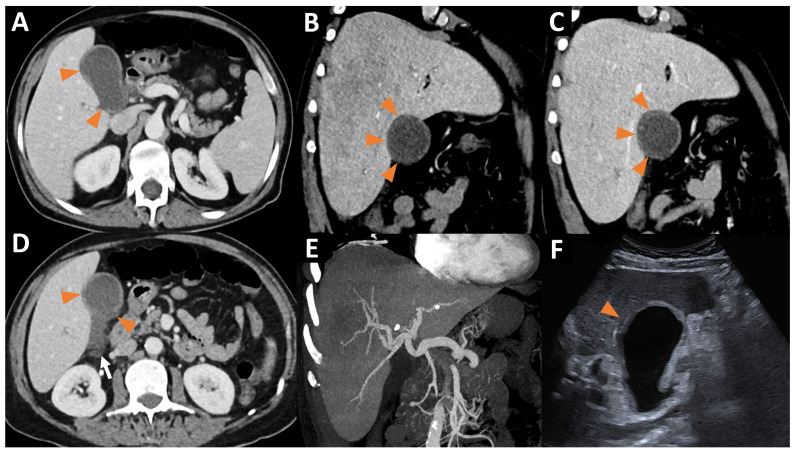
(**A**) Axial CT at the portal phase showing segmental enhancement defects of the gallbladder wall (orange arrowheads). (**B**,**C**) Oblique coronal CT at the arterial (**B**) and portal phase (**C**) showing a segmental enhancement defect of the hepatic side of the gallbladder wall (orange arrowheads). Again, we found that the heterogeneous hepatic enhancement was linked to the arteriovenous shunt between the left branch of the hepatic artery and the left hepatic vein at the arterial phase (**B**). (**D**) Axial CT at the portal phase showing thickening of the gallbladder wall with segmental enhancement defects of the gallbladder wall (orange arrowheads) and a perivesicular fluid effusion (white arrow). (**E**) Coronal CT at the arterial phase with MIP 30 mm, showing a significant enlargement of the hepatic artery. (**F**) Mode B ultrasound showing thickening of the gallbladder wall, with a hypoechoic area of the hepatic side of the gallbladder wall (orange arrowhead), corresponding to the areas of segmental enhancement defects visualized on CT. Ultrasound revealed no gallstones in the gallbladder.

The CT scan revealed a moderate thickening of the gallbladder wall associated with the infiltration of the perivesicular fat and a small perivesicular effusion. A segmental parietal enhancement defect was also noted (Figure 2).

We confirmed these results by an ultrasound exam, which was also suggestive of acalculous cholecystitis (Figure 2).

The diagnosis of ischemic cholecystitis was suspected based on these imaging results, a biological inflammatory syndrome, and a morphine-requiring pain in the right hypochondrium.

A laparoscopic cholecystectomy was performed and revealed a preperforative gallbladder with locally thinned and necrotic walls. The histopathological results confirmed the diagnosis of acute ischemic cholecystitis, without stone.

Additional treatment with antibiotics for 10 days resulted in complete recovery and a safe discharge home.

Since this episode, the patient has been free of abdominal pain and hemoglobin levels have remained normal without blood transfusion or endoscopic treatment.

## 3. Discussion and Literature Review

We report here a very rare case of spontaneous ischemic cholecystitis in the context of HHT.

Cases of ischemic cholecystitis reported in patients with HHT that have been reported so far were only in the context of hepatic artery embolization, performed in symptomatic patients (portal hypertension, heart failure, etc.) [12,13], and this is probably one of the reasons why the embolization of the hepatic artery is no longer recommended according to the international guidelines of 2011 and 2020 [3,14].

Cholangitis due to biliary ischemia is a rare but well-known complication of HHT [8] due to a blood flow steal through arteriovenous shunting and can lead to liver transplantation. Only few ischemic cholangitis cases have been described in the literature [10], and to the best of our knowledge, no case of spontaneous ischemic cholecystitis has been described in patients with HHT.

In this case, the severe episode of epistaxis with significant blood loss (700 mL) has led to a transient decrease in cardiac output [15]. Moreover, the use of norepinephrine to overcome hypovolemia and improve tissue perfusion induced systemic arterial vasoconstriction [16]. These elements, combined with arterial vascular steal due to pre-existing arteriovenous shunts, led to ischemic cholecystitis in this patient with HHT.

A cholecystectomy rather than a cholecystostomy was preferred according the international guidelines for the management of HTT that do not recommend biopsy or transhepatic pathways [14].

## 4. Conclusions

Our case report a spontaneous ischemic cholecystitis in a patient with Hereditary Hemorrhagic Telangiectasia with liver involvement, suspected on imaging and confirmed by surgery and pathology.

## Data Availability

Data are available on reasonable request.
